# Ashwagandha (*Withania somnifera*) and Its Effects on Well-Being—A Review

**DOI:** 10.3390/nu17132143

**Published:** 2025-06-27

**Authors:** Michał Wiciński, Anna Fajkiel-Madajczyk, Józef Sławatycki, Monika Szambelan, Paweł Szyperski, Paweł Wojciechowski, Jakub Wójcicki, Michał Gawryjołek

**Affiliations:** 1 Department of Pharmacology and Therapeutics, Faculty of Medicine, Collegium Medicum in Bydgoszcz, Nicolaus Copernicus University, M. Curie Skłodowskiej 9, 85-094 Bydgoszcz, Poland; anna.fajkiel@cm.umk.pl (A.F.-M.); jozef.slawatycki@cm.umk.pl (J.S.); monika.szambelan@cm.umk.pl (M.S.); pawel.szyperski@cm.umk.pl (P.S.); pawel.wojciechowski@cm.umk.pl (P.W.); jakub.wojcicki@cm.umk.pl (J.W.); 2 Department of Orthopaedics and Traumatology, Dr L. Blazek Multi-Specialty Hospital, 88-100 Inowroclaw, Poland; gawryjolekm83@gmail.com

**Keywords:** ashwagandha, depression, anxiety, stress, sleep quality, cognitive function

## Abstract

In recent decades, the mental health and sleep quality of populations have worsened globally, leading healthcare professionals and researchers to seek new, safe therapies that extend beyond traditional pharmacological treatments. *Withania somnifera*, commonly known as ashwagandha, appears to be a valuable element for improving overall quality of life due to its abundance of active substances and known multidirectional effects on the human body. This review aims to critically evaluate the available literature on ashwagandha extract and its potential roles in enhancing well-being, with a focus on reducing stress, fatigue, anxiety, and depressive disorders; improving sleep quality; and enhancing cognitive function. Furthermore, an attempt has also been made to highlight the limitations associated with the use of this plant extract in clinical practice and indicate directions for future research.

## 1. Introduction

In recent years, the global prevalence of mental health issues and sleep disturbances has increased, leading healthcare providers and the public to seek integrative methods beyond traditional drug therapies. As worries regarding the side effects and shortcomings of usual treatments for mood disorders, anxiety, and insomnia have grown, the quest for effective, evidence-based natural remedies has intensified [1]. One plant that has garnered notable scientific and clinical attention is *Withania somnifera*, also known as ashwagandha.

Ashwagandha is a small shrub native to the Indian subcontinent, the Middle East, and parts of Africa. The name ”ashwagandha” derives from Sanskrit and translates to “smell of a horse,” a reference to its unique aroma and its reputed ability to impart strength and vitality akin to that of a horse. Used for over 3000 years in Ayurvedic medicine, ashwagandha is considered a rasayana—a rejuvenating herb that promotes longevity, resilience to stress, and overall health [2]. It has traditionally been employed to enhance stamina, restore energy, reduce fatigue, and combat stress-related conditions [3,4].

**Figure 1:**
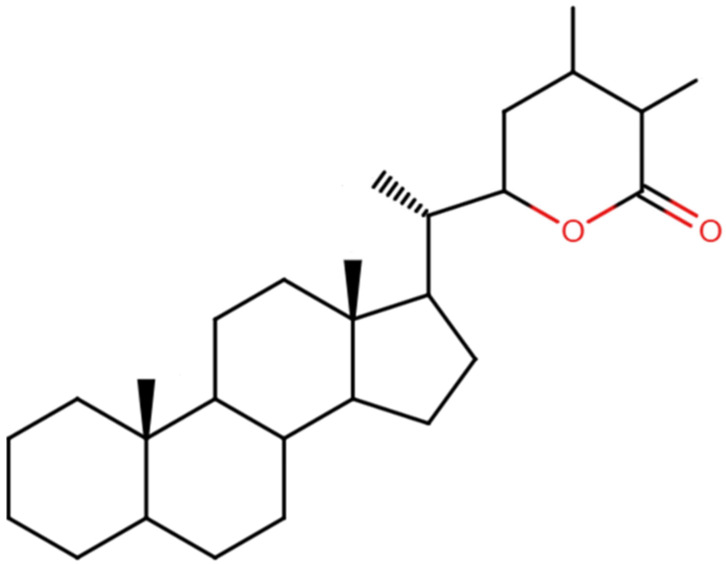
Basic chemical structure of withanolides.

Phytochemically, ashwagandha contains several biologically active constituents that mediate its therapeutic effects. The most studied compounds are withanolides (Figure 1), a group of steroidal lactones that exhibit anti-inflammatory, antioxidant, immunomodulatory, and neuroprotective properties [5]. Other notable components include alkaloids (such as somniferine and tropine), sitoindosides, and acylsterylglucosides, many of which have been suggested to modulate the hypothalamic–pituitary–adrenal (HPA) axis and the sympathetic nervous system [6]. These biochemical effects contribute to ashwagandha’s ability to function as an adaptogen, helping the body to mitigate and adapt to psychological and physical stressors [7].

The global burden of mental health disorders is substantial. According to the World Health Organization (WHO), depression affects over 280 million people worldwide and is a leading cause of disability. Anxiety disorders—including generalized anxiety disorder (GAD), panic disorder, and social phobia—affect an estimated 301 million individuals [8]. In parallel, insomnia and sleep disturbances have become increasingly prevalent in modern societies, often exacerbated by digital overstimulation, work-related stress, and social isolation. Epidemiological studies indicate that acute or transient insomnia affects 30% to 50% of adults, while chronic insomnia—defined as sleep difficulties occurring at least three times per week for three months or more—impacts approximately 10% to 15% of the adult population [9].

These conditions frequently coexist and can profoundly impact the quality of life, work performance, interpersonal relationships, and physical health of individuals. Chronic stress and poor sleep are associated with an increased risk of cardiovascular disease, metabolic syndrome, and immune dysfunction [10,11]. In clinical practice, pharmacotherapy remains the primary treatment modality for mood and sleep disorders. Antidepressants, such as selective serotonin reuptake inhibitors (SSRIs) and serotonin–norepinephrine reuptake inhibitors (SNRIs), alongside benzodiazepines and non-benzodiazepine hypnotics, are commonly prescribed [12]. However, these medications often induce side effects, including sedation, weight gain, sexual dysfunction, dependency, and withdrawal symptoms. Consequently, many individuals choose to discontinue treatment or explore complementary options [13].

In light of these challenges, there has been a significant increase in both public and academic interest in natural health products and herbal medicine. Consumers are increasingly drawn to botanicals that offer therapeutic benefits alongside a safer profile. Ashwagandha has emerged as a leader in this movement, owing to its traditional usage and an expanding body of scientific research that supports its potential psychotropic effects. This review aims to critically evaluate studies on ashwagandha and its potential benefits for overall well-being, specifically in terms of mood regulation, stress relief, sleep enhancement, and cognitive function. PubMed and Google Scholar databases were searched using a combination of keywords: ashwagandha, *Withania somnifera*, stress, depression, anxiety, insomnia, sleep, fatigue, cognitive function, and memory. The search mainly included studies published between 2009 and 2025. The review primarily included in vivo studies, particularly clinical trials.

Through synthesizing the available evidence, we aim to determine whether ashwagandha can serve as a viable adjunct or alternative to standard treatments, while also identifying areas where further research is needed.

## 2. The Effects of Ashwagandha on Stress, Anxiety, and Depression

Stress is currently a widespread symptom that causes deterioration of quality of life. In an evolving world, there has been a rise in psychiatric disorders as well as an escalation in social issues, including substance abuse and related concerns and addictions. An increasing number of individuals are directing their attention towards lifestyle choices and the equilibrium between work and personal life to mitigate stress, anxiety, and depression [14]. We are seeking remedies, psychological support, and pharmacological treatment. Observations of human physiology suggest that the experience of stress is linked to hormonal disorders, primarily within the HPA axis, leading to elevated cortisol levels in the bloodstream [15].

In the study by Lopresti et al. [16], it was shown that the use of ashwagandha at a dosage of 240 mg/day for 60 days led to a reduction in the feeling of stress and fatigue assessed using the Hamilton Anxiety Rating Scale (HAM-A) and the Depression, Anxiety, and Stress Scale-21 (DASS-21). It has also been shown to reduce morning cortisol levels and increase testosterone levels in men, but not in women [16]. Another study evaluated 54 adults with generalized depression [17]. It was shown that administering 500 mg of ashwagandha root extract caused significant improvements in the Perceived Stress Scale (PSS) and GAD-7 scale, a reduction in morning cortisol in saliva, an increase in serotonin in urine, and no effect on nitric oxide (NO), glutathione (GSH), and malondialdehyde (MDA) concentrations [17]. In the study by Baker et al. [18], the experience of stress and coding abilities of students exposed to stress during study were assessed. The study results indicate that a daily dose of 700 mg of ashwagandha enhances sleep, boosts energy, and improves mental clarity, thereby helping to lower stress levels [18]. The researchers observed that ashwagandha in the dose mentioned above had no effect on stress over a 30-day follow-up period. It appears that a more prolonged period of use is necessary to relieve stress [19].

In a study of overweight and obese men, no reduction in stress levels on the PSS was observed compared to the placebo. Interestingly, men showed an increase in free testosterone and luteinizing hormone levels [4]. In a study examining 52 adults who were administered 300 mg of ashwagandha extract daily, it was found that this extract had a beneficial effect on weight management and cortisol reduction in individuals experiencing chronic stress [20]. Another recent study evaluated the effectiveness of various doses in 98 healthy individuals. It examined the 14-item PSS along with stress-related biochemical parameters. Changes in the HPA axis were confirmed, regardless of the dosage [21]. The findings indicate that ashwagandha positively impacts the reduction in stress and anxiety, primarily by altering hormonal levels. The results of the studies discussed above are shown in Table 1.

Arumugam et al. [22] conducted a meta-analysis examining the effects of ashwagandha on stress and anxiety. Their review of studies involving 558 participants revealed a significant effect of ashwagandha, compared to a placebo, on the PSS, Hamilton Anxiety Scale (HAS), and serum cortisol levels. Mild side effects were noted, and the need to evaluate the long-term safety of ashwagandha extract was emphasized [22]. Another systematic review and meta-analysis, which included five clinical trials (254 participants), revealed that ashwagandha significantly reduces HAM-A scores; however, the researchers emphasized the need for studies with larger sample sizes [23].

Given the widespread prevalence of depression, anxiety, and stress in society, it appears that the studies mentioned above were conducted in relatively limited populations. Additionally, some researchers have suggested that the study duration should be extended to adequately observe clinical changes, such as reductions in stress levels. Furthermore, comparative data are deficient regarding the efficacy of ashwagandha extract compared to that of antidepressants and anti-anxiety medications.

## 3. The Effects of Ashwagandha on Fatigue and Sleep Quality

As sleep is a crucial physiological process that allows the human organism to regenerate both physical and mental dimensions, its quality and duration are essential [24]. These factors make insomnia a significant challenge for patients who suffer from it, as it diminishes quality of life due to a lack of proper regeneration and rest. Insomnia is a very common sleep disorder nowadays, and, due to the adverse effects of most available medications, innovative strategies are under consideration by scientists around the world [25]. Furthermore, prevalent high stress and insufficient sleep and rest often lead to pathological fatigue, which can reduce both mental and physical efficiency throughout the day and impact overall quality of life.

Based on their animal study, Kaushik et al. [26] have suggested that ashwagandha extract may positively affect sleep not due to withanolides but rather due to triethylene glycol (TEG), which is found in ashwagandha leaves and may be the most effective component for promoting sleep.

Although the exact molecule responsible for the influence on sleep has not yet been identified, numerous scientific articles, including randomized, double-blinded, prospective studies, confirm ashwagandha’s beneficial effect on physiological processes. Its influence has been observed in individuals aged 18 to 85 [4,24,25,27,28,29]. This age range is significant, as all groups—young adults, adults, and senior citizens—are susceptible to insomnia stemming from various causes. Young adults and adults primarily experience insomnia due to stress, while the elderly are often affected by the aging process, age-related diseases, and pain [29]. The fact that ashwagandha is effective across all age ranges makes it a promising and relatively safe remedy, as noted by Dimpfel et al. [27], who reported some mild side effects. Specifically, one patient experienced an allergic reaction, and two patients (who were treated with hypotensive drugs) reported an increase in blood pressure. The most influential aspects of ashwagandha extract intake include improved sleep quality, waking up refreshed, and enhanced sleep efficiency. Additionally, it reduces the time it takes to fall asleep, increases mental alertness upon waking, and extends total sleep duration [4,24,25,27,28,29,30]. Authors often point out that the improvement is dose-dependent up to a certain point. Salve et al. [28] observed that a 300 mg ashwagandha dose administered twice daily appears to be more effective than a 125 mg dose administered twice daily. Pérez-Piñero et al. [24] compared the effects of different ashwagandha doses with a combination of ashwagandha and tryptophan. The results showed that the 600 mg ashwagandha extract dose had the most significant positive influence on sleep, followed by the 250 mg ashwagandha dose group, which had the second-best effect. In contrast, the combination of 250 mg ashwagandha and tryptophan exhibited the weakest effect, although it still demonstrated improvement compared to the placebo group [24].

Murthy et al. [31] performed a study on animals and cell cultures that presented the molecular influence of ashwagandha—the extract positively affects the gene expression of the ρ1 subfamily of GABAA receptors and histamine type 3 receptors, both in a dose-dependent manner (the gene expression increase was higher in 30 μg/mL than in 15 μg/mL ashwagandha extract arm of the study). The authors also emphasized the dose-dependent reduction in time to fall asleep and the increase in sleep duration in animals [31]. Another study conducted on animals compared the influence of water (WA) and enzyme-treated (EA) ashwagandha root extracts on sleep parameters in mice and rats. Park et al. [32] observed that the enzyme-treated extract—likely due to the removal of starch—resulted in increased sleep duration even at higher doses. In contrast, the water extract demonstrated its peak impact at a 60 mg/kg dose, with no positive change and even a decrease in sleep duration at higher doses. Both extracts reduced sleep latency in a group of pentobarbital-treated mice; however, this effect did not reach statistical significance. In this model, both treatments notably extended sleep duration, with the EA extract proving more effective. On the other hand, in a model of caffeine-induced insomnia, the EA extract significantly reduced sleep latency and increased sleep duration at higher doses (100 and 150 mg/kg) [32].

Fatigue is a problematic condition that reduces human efficiency and involves cognitive and mental impairment. It can result from inadequate sleep, poor sleep quality, or occur independently of these factors. Unfortunately, it is quite common in modern society [33]. Ashwagandha is also regarded as an anti-fatigue agent. This effect is linked to its relaxing, stress-relieving, and sleep-inducing properties [4,33,34,35]. An interesting study in this field was performed by Cooley et al. [36]. They compared ashwagandha extract at a dosage of 300 mg twice daily for 12 weeks with the effects of standard psychotherapy in a group of women and men experiencing moderate to severe anxiety. In this double-blind, randomized trial, ashwagandha showed greater reductions in subjective fatigue, physical fatigue, motivation, and concentration levels compared to conventional psychotherapy [34]. Several studies also emphasize that *W. somnifera* extract has greater anti-fatigue effects than those of the placebo [4,34,35]. However, Lopresti et al. tested ashwagandha extract at a dose of 600 mg in overweight male patients aged 40 to 70, and reported a decrease in fatigue in both the ashwagandha and placebo groups [33]. The results of the studies discussed above are shown in Table 2.

Cheah et al. [37] confirm, in their meta-analysis, the beneficial effect of ashwagandha extract on sleep-related regeneration. Nevertheless, the aforementioned meta-analysis highlights that data on severe adverse effects of ashwagandha extract are limited, and further studies should focus on safety issues related to its long-term use [37].

## 4. The Effects of Ashwagandha on Cognitive Function and Memory

Cognitive functions and memory are fundamental to daily functioning, learning, and decision-making processes. Impairments in cognitive functions and memory can significantly impact a patient’s quality of life and adversely affect overall mental well-being. The pathogenesis of cognitive impairment is multifaceted; however, the primary mechanism involves damage to neuronal tissue. This damage varies according to the underlying cause, which may include aging, psychological stress, brain injury, neurological disorders, and chronic diseases such as hypertension and diabetes [38].

Recent research has highlighted intriguing findings regarding the impact of ashwagandha on cognitive function. Table 3 summarizes these studies.

Remenapp et al. [39] studied 58 healthy subjects aged 18 to 54 who received 225 or 400 mg of ashwagandha extract daily for 30 days in a randomized, double-blind, placebo-controlled clinical trial. In contrast to the placebo group, both ashwagandha groups demonstrated significant improvements in flexibility, complex attention, executive functioning, processing time, and reaction time [39]. Likewise, in a double-blind, randomized, placebo-controlled clinical study, Gopukumar et al. [30] studied 130 healthy, cognitively sound adults aged 20–55 who received 300 mg/day of ashwagandha root extract for 90 days. The results showed a significant improvement in recall memory and a reduction in the total error rate when recalling patterns [30].

In line with the abovementioned studies, Leonard et al. [34] examined 59 healthy participants aged 18 to 60, administering 225 mg of ashwagandha extract daily for 90 days. They have observed improved word recall, choice reaction time, picture recognition, digit vigilance, and the Stroop Color–Word Test [34]. The Stroop Color–Word Test serves as a diagnostic tool for cognitive impairment, requiring the participant to identify the colors printed in a contrasting font. This test checks mainly attention, vigilance, and executive functions [40].

**Table 3:** Summary of research on the effects of ashwagandha on cognitive function and memory.

Characteristics of the Group and Duration of the Study	Daily Doses of Ashwagandha Extract	Effects on Cognitive Function and Memory	Study
130 participants 20–55 years old; 90 days	300 mg	Improvement in recall memory. A decrease in the total error rate in recalling patterns.	[30]
59 participants 18–60 years old; 30 days	225 mg	Improvement in word recall, choice reaction time, picture recognition, digit vigilance, and Stroop Color and Word test.	[34]
58 participants 18–54 years old; 30 days	225 or 400 mg	Improvement in flexibility, complex attention, executive functioning, processing speed, and reaction time.	[39]
120 participants 30–75 years old; 8 weeks	600 mg	Improvement in episodic memory, working memory, and accuracy of attention.	[41]
50 adults; 8 weeks	600 mg	Significant improvements in immediate and general memory, executive function, sustained attention, and information processing speed.	[42]
40 participants with MCI; 60 days	225 mg	Improvement in immediate memory, general memory, working memory, and visuospatial processing. Increase in MMSE scale.	[43]

Kale et al. [41] reported similar effects in a prospective, randomized, placebo-controlled study in which 120 healthy subjects took 600 mg of ashwagandha root extract daily for 8 weeks. The extract was well tolerated and reported no clinically significant side effects [41].

Another study highlighting the improvement of cognitive functions was conducted by Choudhary et al. [42], who provided 50 participants with mild cognitive impairment (MCI) with 300 mg of ashwagandha root extract twice daily for 8 weeks. Significant improvements were observed in immediate and general memory, executive function, sustained attention, and information processing speed [42].

Rai et al.’s [43] study reported very interesting outcomes. They studied 40 patients with MCI who received 225 mg/day of ashwagandha extract for 60 days. They observed improvements in immediate memory, general memory, working memory, and visuospatial processing, as well as an increase in the Mini-Mental State Examination (MMSE) scale [43]. This scale is a screening test for cognitive impairment, primarily used in elderly individuals. It assesses the main cognitive domains, including orientation, registration, attention, recall, language, and visuospatial skills [44].

Several attempts have been made to elucidate the potential mechanism of action of ashwagandha extracts in human cells. Kuboyama et al. investigated in vitro that withanolide A isolated from ashwagandha root extract regenerates neurites and recovers synapses in heavily damaged mouse neurons [45]. Another study by Kuboyama et al. shows that withanolide A, IV, and VI are responsible for enhancing the outgrowth of human neuroblastoma SH-SY5Y dendrites and axons [46]. These studies indicate the regenerative effect of ashwagandha extract on nerve cells. Secondarily, it may improve the neural transmission between synapses, which is beneficial for patients with cognitive impairment. Unfortunately, the exact mechanism by which ashwagandha improves cognitive function and memory is still unknown.

According to the aforementioned studies, supplementation with ashwagandha extract appears beneficial for individuals in good health and for patients suffering from cognitive impairment. It improves daily functioning, memory, and visuospatial functioning, reduces stress, and improves sleep quality. The recommended daily dosage ranges from 225 to 600 mg. The daily dosage and length of therapy do not notably influence the outcomes achieved. Importantly, no significant adverse effects were observed during the trials in any of the study groups. On the other hand, the studies mentioned above are characterized by the limited size of the participant groups and the brief duration of the investigations. Furthermore, all studies evaluated the efficacy of ashwagandha extract in comparison to a placebo, thereby rendering it infeasible to conclude its effectiveness relative to standard pharmacotherapy for cognitive impairment.

## 5. Limitations

The studies discussed in the chapters above indicate that ashwagandha can be a valuable supplement for improving mood and sleep quality, reducing fatigue and stress, and improving memory and cognitive function. Nevertheless, discussing its potential contraindications as a stand-alone therapeutic agent and an adjunct to standard treatment seems necessary. Regarding the aforementioned studies, it is essential to note the small number of participants involved. Moreover, many studies were conducted over a limited duration (ranging from 30 days to a maximum of 16 weeks), which may have failed to demonstrate the complete clinical effect. This relatively brief duration of research may also inadequately reflect the impact of prolonged use of ashwagandha extract, particularly in instances of self-medication. Interestingly, in most studies, ashwagandha’s efficacy has been compared to a placebo, so data comparing its effects with standard pharmacotherapy (e.g., SSRIs, hypnotics) are lacking. It appears to be a valid approach to closely examine the potential dangers associated with the unregulated use of this plant material.

Most studies suggest that the recommended dose of ashwagandha is 250–600 mg/day [28]. The most common and popular form of ashwagandha is powder, which contains the herb’s dried root, leaf, or combination [47]. The preparations with ashwagandha extract available on the pharmaceutical market are mainly dietary supplements, which simplifies registration procedures and may impact the quality of the available products [48]. Some studies have used sustained-release capsules containing ashwagandha extract, which may be associated with increased bioavailability and, consequently, improved utilization of the active ingredients contained in this plant [30]. Moreover, due to the wide range of phytochemicals found in ashwagandha, it is crucial to develop a suitable analytical method to measure the various withanolides in ashwagandha, allowing its extracts to be standardized [49].

The clinical trial on the safety of ashwagandha showed that supplementation with ashwagandha root extract at 300 mg/day for 8 weeks demonstrated no toxic effects in healthy individuals [50]. In contrast, there are increasing reports of potential liver-damaging effects, which are even referred to as ashwagandha-induced cholestatic hepatitis. This is particularly dangerous for patients with pre-diagnosed liver disease and can even lead to acute and chronic liver failure syndrome [51,52,53]. Björnsson et al. [52] detailed a case involving five patients who experienced jaundice, nausea, pruritus, and abdominal discomfort after using ashwagandha preparations for 2 to 12 weeks. The average age of the patients was 43 years, with three being men. The findings indicated that the liver damage was cholestatic or mixed, although none of the patients experienced liver failure. Notably, in one patient who was also taking *Rhodiola rosea*, a potential herb–herb interaction was suspected to have contributed to the liver damage [52]. This case highlights the possible dangers of concomitant use with other adaptogens. Another reported case involved a 39-year-old woman who presented with a week-long history of jaundice and nausea after taking a supplement containing ashwagandha root extract. The patient displayed abnormalities in liver enzymes linked to jaundice, and a liver biopsy showed acute cholestatic hepatitis with subepithelial necrosis; however, no features indicating chronicity were observed [52]. Additional cases of herb-induced liver injury (HILI) involve a man and a woman who purchased ashwagandha supplements online to boost fertility [54]. A 36-year-old male took ashwagandha extract at a dosage of 450 mg three times a day for six months. His symptoms included nausea, itching, and dark urine. In contrast, a 30-year-old female took 450 mg of ashwagandha daily and experienced itching after 45 days. In both cases, there were elevated levels of serum bilirubin and liver enzymes, specifically aspartate transaminase (AST), alanine transaminase (ALT), and alkaline phosphatase (ALP). After stopping ashwagandha supplements, both individuals showed improvements in their clinical condition and liver function [54].

Not all patients are suitable candidates for ashwagandha. Those with hyperthyroidism who present symptoms like irritability, restlessness, anxiety, hand tremors, palpitations, psychomotor agitation, muscle weakness, fatigue, and reduced libido fall into this category. While products containing ashwagandha root extract have demonstrated effectiveness in alleviating these symptoms, their use is contraindicated for individuals with hyperthyroidism, as they can worsen the condition. Research indicates that this raw material can raise the levels of 3, 3′, 5-triiodothyronine (T3) and tetraiodothyronine (T4), which is detrimental for those suffering from hyperthyroidism [55]. An illustrative example of a perilous trend related to self-medication with ashwagandha is the case of a 73-year-old woman who experienced supraventricular tachycardia, hyperthyroid symptoms, and markedly low thyroid-stimulating hormone (TSH) levels following two years of utilizing ashwagandha extract as a self-treatment for hypothyroidism. Discontinuing the supplement resulted in complete symptom resolution and enhanced biochemical markers [55]. Ashwagandha root extract is known for treating male infertility, but it is not recommended for men with hormone-sensitive prostate cancer since it may boost testosterone production, accelerating disease progression. Additionally, ashwagandha is contraindicated for those who are pregnant or planning to conceive, as high doses of ashwagandha root extract could result in miscarriage [56,57].

Interactions with other drugs also remain an important issue. Due to its synergistic properties, ashwagandha root extract may enhance the effects of anti-anxiety, sleep, muscle-relaxant, and sedative medications. This ingredient shows additive effects with anticonvulsants, barbiturates, and benzodiazepines, potentially leading to heightened adverse reactions, including impaired motor coordination, muscle weakness, headaches, reduced libido, muscle tremors, and drowsiness [58]. Analysis of herb–drug interactions has shown that the concomitant use of ashwagandha with antidepressants can lead to several dangerous side effects. Interaction with reboxetine may result in testicle pain and ejaculatory dysfunction, while interaction with sertraline can cause severe diarrhea. Additionally, combining ashwagandha with escitalopram can lead to myalgia, epigastric pain, nausea, vomiting, restless legs syndrome, and a severe cough. In contrast, the combination with paroxetine may result in generalized myalgia, ophthalmalgia, and ocular hypertension. From a pharmacokinetics perspective, the inhibition of CYP3A4 and CYP2D6 by ashwagandha increases the concentration and side effects of antidepressants that are metabolized by these cytochromes [59].

It seems that ashwagandha may not be suitable for everyone, and potential side effects should be taken into account when selecting such a supplement, especially for elderly patients and those taking other medications. Additionally, there is a lack of substantial data regarding the long-term effects of ashwagandha extract use and its safety. Moreover, researchers face the challenge of standardizing ashwagandha formulations, which is inherently linked to the quality of the preparations used.

## 6. Conclusions

At present, due to stress and overwork, mood and sleep disorders are affecting an increasing number of people. Hence, natural remedies with proven effects are being actively searched for. Ashwagandha is a plant that covers many needs, not only related to mental health but also somatic health. Studies have proven its positive effects in terms of reducing stress and fatigue using objective scales (HAM-A, DASS-21) and in laboratory tests, such as lowering morning cortisol levels and increasing testosterone levels in men. Modulation of the HPA axis and the amelioration of neuronal transmission between synapses contribute to the reduction in anxiety and insomnia, thereby improving cognitive ability and overall mental health.

Most studies have used ashwagandha root extract, while others have used leaves or both. Depending on the expected effect, it is essential to systematize the composition to maximize the result. This will confirm the positive impact of a specific ingredient in an exact dose for a particular ailment (e.g., TEG contained in leaves and its effect on insomnia). Approving ashwagandha as a drug or medical device would enable better control over the preparations available for use by researchers and patients. The high quality of preparations containing ashwagandha extract, coupled with an in-depth examination of side effects and the specific patient groups that may experience adverse reactions, is a significant concern that requires further investigation. Moreover, when recommending ashwagandha extract, it is essential to consider the patient’s medical condition and any potential interactions with other medications they may be taking.

Future research on ashwagandha extract should focus on standardizing formulation protocols, long-term safety trials, and testing on vulnerable populations, especially the poly-treated elderly and those with endocrine disorders. Additionally, mechanistic studies using omics and neuroimaging tools may also provide valuable scientific insights.

## Figures and Tables

**Table 1:** Summary of research on the effects of ashwagandha on stress, anxiety, and depression.

Characteristics of the Group and Duration of the Study	Daily Doses of Ashwagandha Extract	Effect on Stress, Anxiety, and Depression	Study
60 stressed, healthy adults; 60 days	240 mg	Reducing feelings of anxiety and stress.	[16]
54 stressed, healthy adults; 60 days	500 mg	Reducing feelings of anxiety and stress.	[17]
60 healthy college students aged 18–50; 30 days	700 mg	Enhancing sleep, boosting energy, and improving mental clarity. No effect on stress over 30 days of follow-up.	[18] [19]
120 overweight or mildly obese men and women aged 40–75 years; 12 weeks	400 mg	No reduction in the level of stress.	[4]
52 adults aged 18–60; 8 weeks	300 mg	Stress reduction, weight loss, and cortisol level reduction.	[20]
98 adults; 8 weeks	125, 250, and 500 mg	Reduction in mild to moderate stress regardless of dose.	[21]

**Table 2:** Summary of research on the effects of ashwagandha on fatigue and sleep quality.

Characteristics of the Group and Duration of the Study	Daily Doses of Ashwagandha Extract	Effect on Sleep	Effect on Fatigue	Study
120 overweight or mildly obese men and women aged 40–75 years; 12 weeks	400 mg	Quality increased in both groups.	A larger decrease in the ashwagandha group and a smaller decrease in the placebo group.	[4]
59 men and women aged 18–49 years; 30 days	225 mg	N/A	There was a small decrease in the ashwagandha group.	[34]
57 overweight men aged 40–70 years; 16 weeks	600 mg	N/A	Improvement in both groups with no statistically significant difference.	[33]
13 women and men aged 18–59 years; 4–7 days	400 mg	N/A	Less mental fatigue in tests in the ashwagandha group.	[35]
16 women and men aged 60–75 years; 4 weeks	300 mg	Increase in sleep quality and refreshment after sleep.	N/A	[27]
41 employees with moderate to severe anxiety; 12 weeks	600 mg	N/A	A decrease in physique and subjective fatigue in the ashwagandha group compared to psychotherapy.	[36]
60 women and men aged 18–55 years; 8 weeks	250 mg or 600 mg	Increase in sleep quality in ashwagandha groups, with more effectiveness in the ashwagandha 600 mg/day group.	N/A	[28]
Mice and rats; 4 weeks	Ashwagandha root water extract (AW) vs. enzyme-treated ashwagandha root extract (EA)	Decrease in sleep latency—statistically insignificant for AW and EA. Sleep duration increases with peak performance in the 60 mg/kg dose for AW, while sleep duration increases for EA even in higher doses (60, 80, 100, and 150 mg/kg). Decreased sleep latency and increased sleep duration in caffeine-induced insomnia mice for EA.	N/A	[32]
130 women and men aged 20–55 years; 90 days	300 mg	Increase in sleep quality.	N/A	[30]
C6 rat glioma-established cell line and Swiss albino male mice	15 mg/ml or 30 mg/ml	Increase in GABA_A_ρ1 and H3 receptor gene expression in a dose-dependent manner. Sleep latency decreases and sleep time increases in a dose-dependent manner.	N/A	[31]
60 women and men aged 18–60 years suffering from insomnia; 10 weeks	600 mg	Decrease in sleep latency and wake after sleep onset. Increase sleep efficiency, total time in bed, total sleep time, and subjective sleep quality.	N/A	[25]
52 young adults of both genders; 90 days	250 mg or 250 mg + tryptophan or 600 mg	Sleep quality improvement from the highest to the lowest: ashwagandha 600 mg group, ashwagandha 250 mg group, ashwagandha 250 mg + tryptophan 175 mg group, and placebo group.	N/A	[24]
39 women and men aged 60–85 years; 12 weeks	600 mg	An increase in sleep quality and mental alertness in the ashwagandha group.	N/A	[29]
Male C57BL/6 mice with EEG monitoring	Alcohol-based ashwagandha leaf extract vs. water-based ashwagandha leaf extract vs. cyclodextrin-assisted aqueous ashwagandha leaf extract	No influence on REM and NREM sleep phases in alcohol extract; NREM duration increases in water and cyclodextrin extracts.	N/A	[26]

N/A—not available.

## References

[1] Urbanska N., Ashaolu T.J., Mattova S., Simko P., Kiskova T. (2025). The Potential of Selected Plants and Their Biologically Active Molecules in the Treatment of Depression and Anxiety Disorders. Int. J. Mol. Sci..

[2] Singh N., Bhalla M., de Jager P., Gilca M. (2011). An overview on ashwagandha: A Rasayana (rejuvenator) of Ayurveda. Afr. J. Tradit. Complement. Altern. Med..

[3] Lee D.-H., Ahn J., Jang Y.-J., Seo H.-D., Ha T.-Y., Kim M.J., Huh Y.H., Jung C.H. (2020). *Withania somnifera* Extract Enhances Energy Expenditure via Improving Mitochondrial Function in Adipose Tissue and Skeletal Muscle. Nutrients.

[4] Smith S.J., Lopresti A.L., Fairchild T.J. (2023). Exploring the efficacy and safety of a novel standardized ashwagandha (*Withania somnifera*) root extract (Witholytin^®^) in adults experiencing high stress and fatigue in a randomized, double-blind, placebo-controlled trial. J. Psychopharmacol..

[5] Sun G.Y., Li R., Cui J., Hannink M., Gu Z., Fritsche K.L., Lubahn D.B., Simonyi A. (2016). *Withania somnifera* and Its Withanolides Attenuate Oxidative and Inflammatory Responses and Up-Regulate Antioxidant Responses in BV-2 Microglial Cells. Neuromol. Med..

[6] Wiciński M., Fajkiel-Madajczyk A., Kurant Z., Kurant D., Gryczka K., Falkowski M., Wiśniewska M., Słupski M., Ohla J., Zabrzyński J. (2023). Can Ashwagandha Benefit the Endocrine System?—A Review. Int. J. Mol. Sci..

[7] Mikulska P., Malinowska M., Ignacyk M., Szustowski P., Nowak J., Pesta K., Szeląg M., Szklanny D., Judasz E., Kaczmarek G. (2023). Ashwagandha (*Withania somnifera*)—Current Research on the Health-Promoting Activities: A Narrative Review. Pharmaceutics.

[8] Cuijpers P., Javed A., Bhui K. (2023). The WHO World Mental Health Report: A call for action. Br. J. Psychiatry.

[9] Morin C.M., Benca R. (2012). Chronic insomnia. Lancet.

[10] Lechat B., Appleton S., Melaku Y.A., Hansen K., McEvoy R.D., Adams R., Catcheside P., Lack L., Eckert D.J., Sweetman A. (2022). Comorbid insomnia and sleep apnoea is associated with all-cause mortality. Eur. Respir. J..

[11] Sejbuk M., Mirończuk-Chodakowska I., Witkowska A.M. (2022). Sleep Quality: A Narrative Review on Nutrition, Stimulants, and Physical Activity as Important Factors. Nutrients.

[12] Szuhany K.L., Simon N.M. (2022). Anxiety Disorders: A Review. JAMA.

[13] Khawam E.A., Laurencic G., Malone D.A. (2006). Side effects of antidepressants: An overview. Cleve Clin. J. Med..

[14] Sprung J.M., Rogers A. (2021). Work-life balance as a predictor of college student anxiety and depression. J. Am. Coll. Health.

[15] Stephens M.A., Wand G. (2012). Stress and the HPA axis: Role of glucocorticoids in alcohol dependence. Alcohol. Res..

[16] Lopresti A.L., Smith S.J., Malvi H., Kodgule R. (2019). An investigation into the stress-relieving and pharmacological actions of an ashwagandha (*Withania somnifera*) extract: A randomized, double-blind, placebo-controlled study. Medicine.

[17] Majeed M., Nagabhushanam K., Mundkur L. (2023). A standardized Ashwagandha root extract alleviates stress, anxiety, and improves quality of life in healthy adults by modulating stress hormones: Results from a randomized, double-blind, placebo-controlled study. Medicine.

[18] Baker C., Kirby J.B., O’Connor J., Lindsay K.G., Hutchins A., Harris M. (2022). The Perceived Impact of Ashwagandha on Stress, Sleep Quality, Energy, and Mental Clarity for College Students: Qualitative Analysis of a Double-Blind Randomized Control Trial. J. Med. Food.

[19] O’Connor J., Lindsay K., Baker C., Kirby J., Hutchins A., Harris M. (2022). The Impact of Ashwagandha on Stress, Sleep Quality, and Food Cravings in College Students: Quantitative Analysis of a Double-Blind Randomized Control Trial. J. Med. Food.

[20] Choudhary D., Bhattacharyya S., Joshi K. (2017). Body Weight Management in Adults Under Chronic Stress Through Treatment With Ashwagandha Root Extract: A Double-Blind, Randomized, Placebo-Controlled Trial. J. Evid. Based Complement. Altern. Med..

[21] Pandit S., Srivastav A.K., Sur T.K., Chaudhuri S., Wang Y., Biswas T.K. (2024). Effects of *Withania somnifera* Extract in Chronically Stressed Adults: A Randomized Controlled Trial. Nutrients.

[22] Arumugam V., Vijayakumar V., Balakrishnan A., BBhandari R., Boopalan D., Ponnurangam R., Sankaralingam Thirupathy V., Kuppusamy M. (2024). Effects of Ashwagandha (Withania Somnifera) on stress and anxiety: A systematic review and meta-analysis. Explore (NY).

[23] Fatima K., Malik J., Muskan F., Raza G., Waseem A., Shahid H., Jaffery S.F., Khan U., Zaheer M.K., Shaikh Y. (2024). Safety and efficacy of *Withania somnifera* for anxiety and insomnia: Systematic review and meta-analysis. Hum. Psychopharmacol..

[24] Pérez-Piñero S., Muñoz-Carrillo J.C., Echepare-Taberna J., Muñoz-Cámara M., Herrera-Fernández C., Ávila-Gandía V., Heres Fernández Ladreda M., Menéndez Martínez J., López-Román F.J. (2024). Effectiveness of Enriched Milk with Ashwagandha Extract and Tryptophan for Improving Subjective Sleep Quality in Adults with Sleep Problems: A Randomized Double-Blind Controlled Trial. Clocks Sleep.

[25] Langade D., Kanchi S., Salve J., Debnath K., Ambegaokar D. (2019). Efficacy and Safety of Ashwagandha (*Withania somnifera*) Root Extract in Insomnia and Anxiety: A Double-blind, Randomized, Placebo-controlled Study. Cureus.

[26] Kaushik M.K., Kaul S.C., Wadhwa R., Yanagisawa M., Urade Y. (2017). Triethylene glycol, an active component of Ashwagandha (*Withania somnifera*) leaves, is responsible for sleep induction. PLoS ONE.

[27] Dimpfel W., Schombert L., Keplinger-Dimpfel I.K., Panossian A. (2020). Effects of an Adaptogenic Extract on Electrical Activity of the Brain in Elderly Subjects with Mild Cognitive Impairment: A Randomized, Double-Blind, Placebo-Controlled, Two-Armed Cross-Over Study. Pharmaceuticals.

[28] Salve J., Pate S., Debnath K., Langade D. (2019). Adaptogenic and Anxiolytic Effects of Ashwagandha Root Extract in Healthy Adults: A Double-blind, Randomized, Placebo-controlled Clinical Study. Cureus.

[29] Kelgane S.B., Salve J., Sampara P., Debnath K. (2020). Efficacy and Tolerability of Ashwagandha Root Extract in the Elderly for Improvement of General Well-being and Sleep: A Prospective, Randomized, Double-blind, Placebo-controlled Study. Cureus.

[30] Gopukumar K., Thanawala S., Somepalli V., Rao T.S.S., Thamatam V.B., Chauhan S. (2021). Efficacy and Safety of Ashwagandha Root Extract on Cognitive Functions in Healthy, Stressed Adults: A Randomized, Double-Blind, Placebo-Controlled Study. Evid. Based Complement. Altern. Med..

[31] Murthy S.V., Fathima S.N., Mote R. (2022). Hydroalcoholic Extract of Ashwagandha Improves Sleep by Modulating GABA/Histamine Receptors and EEG Slow-Wave Pattern in In Vitro—In Vivo Experimental Models. Prev. Nutr. Food Sci..

[32] Park C.W., Hong K.B., Suh H.J., Ahn Y. (2023). Sleep-promoting activity of amylase-treated Ashwagandha (*Withania somnifera* L. Dunal) root extract via GABA receptors. J. Food Drug Anal..

[33] Lopresti A.L., Drummond P.D., Smith S.J. (2019). A Randomized, Double-Blind, Placebo-Controlled, Crossover Study Examining the Hormonal and Vitality Effects of Ashwagandha (*Withania somnifera*) in Aging, Overweight Males. Am. J. Mens. Health.

[34] Leonard M., Dickerson B., Estes L., Gonzalez D.E., Jenkins V., Johnson S., Xing D., Yoo C., Ko J., Purpura M. (2024). Acute and Repeated Ashwagandha Supplementation Improves Markers of Cognitive Function and Mood. Nutrients.

[35] Xing D., Yoo C., Gonzalez D., Jenkins V., Nottingham K., Dickerson B., Leonard M., Ko J., Faries M., Kephart W. (2022). Effects of Acute Ashwagandha Ingestion on Cognitive Function. Int. J. Environ. Res. Public Health.

[36] Cooley K., Szczurko O., Perri D., Mills E.J., Bernhardt B., Zhou Q., Seely D. (2009). Naturopathic care for anxiety: A randomized controlled trial ISRCTN78958974. PLoS ONE.

[37] Cheah K.L., Norhayati M.N., Husniati Yaacob L., Abdul Rahman R. (2021). Effect of Ashwagandha (*Withania somnifera*) extract on sleep: A systematic review and meta-analysis. PLoS ONE.

[38] Agrawal S., Schneider J.A. (2022). Vascular pathology and pathogenesis of cognitive impairment and dementia in older adults. Cereb. Circ. Cogn. Behav..

[39] Remenapp A., Coyle K., Orange T., Lynch T., Hooper D., Hooper S., Conway K., Hausenblas H.A. (2022). Efficacy of *Withania somnifera* supplementation on adult’s cognition and mood. J. Ayurveda Integr. Med..

[40] Uttl B., Graf P. (1997). Color-Word Stroop test performance across the adult life span. J. Clin. Exp. Neuropsychol..

[41] Kale S., Lopresti A., Suri R., Garg N., Langade D. (2024). Safety and Efficacy of Ashwagandha Root Extract on Cognition, Energy and Mood Problems in Adults: Prospective, Randomized, Placebo-Controlled Study. J. Psychoact. Drugs.

[42] Choudhary D., Bhattacharyya S., Bose S. (2017). Efficacy and Safety of Ashwagandha (*Withania somnifera* (L.) Dunal) Root Extract in Improving Memory and Cognitive Functions. J. Diet. Suppl..

[43] Rai H.P., Mishra D.N. (2025). Effect of ashwagandha (*Withania somnifera*) extract with Sominone (Somin-On™) to improve memory in adults with mild cognitive impairment: A randomized, double-blind, placebo-controlled study. J. Psychopharmacol..

[44] Arevalo-Rodriguez I., Smailagic N., Roqué-Figuls M., Ciapponi A., Sanchez-Perez E., Giannakou A., Pedraza O.L., Bonfill Cosp X., Cullum S. (2021). Mini-Mental State Examination (MMSE) for the early detection of dementia in people with mild cognitive impairment (MCI). Cochrane Database Syst. Rev..

[45] Kuboyama T., Tohda C., Komatsu K. (2005). Neuritic regeneration and synaptic reconstruction induced by withanolide A. Br. J. Pharmacol..

[46] Kuboyama T., Tohda C., Zhao J., Nakamura N., Hattori M., Komatsu K. (2002). Axon- or dendrite-predominant outgrowth induced by constituents from Ashwagandha. Neuroreport.

[47] Amritha N., Bhooma V., Parani M. (2020). Authentication of the market samples of Ashwagandha by DNA barcoding reveals that powders are significantly more adulterated than roots. J. Ethnopharmacol..

[48] Vazirani S., Kothari A., Fujimoto J., Gomez M. (2023). Supplements Are Not a Synonym for Safe: Suspected Liver Injury From Ashwagandha. Fed. Pr..

[49] Antony B., Benny M., Kuruvilla B.T., Sebastian A., Aravindakshan Pillai A.A., Joseph B., Edappattu Chandran S. (2020). Development and validation of an RP-HPLC method for the simultaneous determination of total withanolide glycosides and Withaferin A in *Withania somnifera* (Ashwagandha). Curr. Chromatogr..

[50] Verma N., Gupta S.K., Tiwari S., Mishra A.K. (2021). Safety of Ashwagandha Root Extract: A Randomized, Placebo-Controlled, study in Healthy Volunteers. Complement. Ther. Med..

[51] Philips C.A., Ahamed R., Rajesh S., George T., Mohanan M., Augustine P. (2020). Comprehensive review of hepatotoxicity associated with traditional Indian Ayurvedic herbs. World J. Hepatol..

[52] Björnsson H.K., Björnsson E.S., Avula B., Khan I.A., Jonasson J.G., Ghabril M., Hayashi P.H., Navarro V. (2020). Ashwagandha-induced liver injury: A case series from Iceland and the US drug-induced liver injury network. Liver Int..

[53] Ireland P.J., Hardy T., Burt A.D., Donnelly M.C. (2021). Drug-induced hepatocellular injury due to herbal supplement Ashwagandha. J. R. Coll. Physicians Edinb..

[54] Bokan G., Glamočanin T., Mavija Z., Vidović B., Stojanović A., Björnsson E.S., Vučić V. (2023). Herb-Induced Liver Injury by Ayurvedic Ashwagandha as Assessed for Causality by the Updated RUCAM: An Emerging Cause. Pharmaceuticals.

[55] Kamal H.I., Patel K., Brdak A., Heffernan J., Ahmad N. (2022). Ashwagandha as a Unique Cause of Thyrotoxicosis Presenting With Supraventricular Tachycardia. Cureus.

[56] Brar G.K., Malhotra M. (2021). Ashwagandha (*Withania somnifera*)—A herb with versatile medicinal properties empowering human physical and mental health. J. Pre-Clin. Clin. Res..

[57] Połumackanycz M., Forencewicz A., Wesołowski M., Viapiana A. (2020). Ashwagandha (*Withania somnifera* L.)—The plant with proven health-promoting properties. Farm. Pol..

[58] Akhgarjand C., Asoudeh F., Bagheri A., Kalantar Z., Vahabi Z., Shab-bidar S., Rezvani H., Djafarian K. (2022). Does Ashwagandha supplementation have a beneficial effect on the management of anxiety and stress? A systematic review and meta-analysis of randomized controlled trials. Phyther. Res..

[59] Siwek M., Woroń J., Wrzosek A., Gupało J., Chrobak A.A. (2023). Harder, better, faster, stronger? Retrospective chart review of adverse events of interactions between adaptogens and antidepressant drugs. Front. Pharmacol..

